# A systematic review of literature on substance use in nightlife settings utilizing *in situ* data collection

**DOI:** 10.1016/j.dadr.2025.100387

**Published:** 2025-10-08

**Authors:** Renata Glavak-Tkalić, Mike Vuolo, Anja Wertag

**Affiliations:** a Ivo Pilar Institute of Social Sciences, Marulićev trg 19, Zagreb 10000, Croatia; b Ohio State University, 268 Townshend Hall, 1885 Neil Avenue Mall, Columbus, OH, United States

**Keywords:** Nightlife, *in situ* research, Substance use, Systematic review, Biomarkers

## Abstract

**Background:**

Nightlife environments, including nightclubs, bars, and entertainment districts, are associated with elevated substance use and related harms. *In situ* nightlife studies offer an opportunity to capture real-time data on substance use from targeted populations. Despite the growing number of studies, no systematic review has yet been conducted on this topic. Therefore, the aim of this systematic review is to explore empirical *in situ* research in nightlife settings, with a focus on substance use.

**Methods:**

A systematic search was conducted across four databases (WOS, PsycInfo, PubMed, and Google Scholar) for English-language peer-reviewed journal articles published between 2014 and 2023 that involved *in situ* primary data collection about substance use in nightlife settings. In total, 55 articles met the inclusion criteria. Detailed data were extracted on various aspects, such as study design, recruitment methods, substances reported, and key findings.

**Results:**

Included studies represented the United States, Europe, Brazil, and Oceania. Most (93 %) employed surveys; over half (56 %) also collected biomarkers. Substance use was highest among males, young adults, and sexual minorities, with polydrug use and high-risk behavior particularly prevalent in Electronic Dance Music scenes. Included articles varied substantially in their focus, including prevalence, correlates, patterns, harms, and interventions. Recruitment and reporting methods varied widely, complicating cross-study comparisons.

**Conclusions:**

This review highlights both the value and challenges of *in situ* research. Biomarker data enhance the reliability of self-report measures, while inconsistent reporting and non-random sampling limit generalizability. Future research should adopt standardized reporting guidelines that would allow for stronger evidence, permit reproducibility, and increase transparency.

## Highlights

• Represents the first systematic review of *in situ* nightlife substance use studies.
• A considerable variety of substances and research questions were noted.
• Demonstrates benefits of real-time data collection, including biomarkers.
• Reveals challenges related to sampling, participation, and reporting.
• Recommend expert-led panel to develop *in situ* nightlife publishing guidelines.

## 1 Introduction

Nightlife environments, including nightclubs, bars, concert venues, and music festivals, are an integral part of contemporary life, attracting many people, particularly young adults (Tutenges, 2021). These settings provide an outlet for relaxation, opportunities for socializing and engaging in diverse experiences; however, they are also associated with a range of health and safety concerns, particularly in relation to substance misuse and other risky behaviors (e.g., Feltmann et al., 2021; Quigg et al., 2020, van Amsterdam et al., 2020). The risks of substance use in nightlife are not uniform but vary across substances, patterns of use, and individual-levelcharacteristics. For example, alcohol use is consistently associated with violence, injuries and impaired driving (World Health Organization, 2024, Calafat et al., 2009); stimulant use, such as MDMA or cocaine, carries risk of dehydration, overheating, and cardiovascular episodes(Parrott, 2013, van Amsterdam et al., 2021), while cannabis use has been associated with impaired memory, attention, and driving performance (Gowin et al., 2025, Volkow et al., 2016).

The most effective way to gain insight into substance use and its associated risks among nightlife patrons, a population that is typically a small subset of general population surveys, is to conduct research *in situ* within nightlife environments (European Monitoring Centre for Drugs and Drug Addiction, 2018). Therefore, in this systematic review, we specifically examine nightlife studies of substance use in which data were collected *in situ* [literally “in place”]. That is, the data were collected in nightlife scenes, either inside venues, immediately outside venues, or in a nightlife area. In what follows, we briefly summarize research on nightlife studies of substance use before turning to our systematic review.

Previous studies emphasize the complex combination of psychological, social, and environmental factors that contribute to the risks associated with substance use in nightlife environments. These environments offer opportunities for the initiation of alcohol and drug use, providing access to and exposure to psychoactive substances (e.g., Bourdeau et al., 2017; Hesse et al., 2010). Alcohol availability and social norms may drive excessive drinking (Dimova et al., 2023), while stimulants like MDMA are often used to enhance sensory and social experiences (Parrott, 2013). Additional factors also contribute to these risks, such as environmental and contextual factors related to nightlife settings (e.g., local regulations, nightlife policies, alcohol sale strategies, staff training) (Bourdeau et al., 2017, Carlini and Sanchez, 2018, Quigg et al., 2018), as well as frequency of attending nightlife venues (Labhart et al., 2020, Sañudo et al., 2015). *In situ* studies provide the opportunity for researchers to observe these environmental factors.

Substance use in nightlife environments is influenced by socio-demographic factors such as age and sex, with studies showing that younger individuals and males are more prone to substance use in these environments (Feltmann et al., 2021; Jørgenrud et al., 2021). Attitudes and motivation for substance use also play an important role in their use. In nightlife environments, the climate toward alcohol and drug use tends to be more tolerant (Boiler et al., 2011), as nightlife patrons may have less negative attitudes towards substance use (Hughes et al., 2019). An integrative literature review by Lojszczyk et al. (2023) showed that a variety of factors motivate drug use as well as alcohol consumption in social settings, which are mostly associated with the desire for positive intoxication effects and novel experiences, such as achieving euphoria, emotional intimacy, social benefits, peer influence, increased confidence, and reduced inhibitions.

Studies also suggest that certain social circles and subcultures are more susceptible to substance use, such as young adults, frequent clubgoers (‘clubbers’), and LGBTQ+  populations (Florêncio, 2023, Herzig and Bachmann, 2017, Palamar et al., 2018). Many studies focus on drug use within the club scene, suggesting that the environments surrounding specific music genres can impact the likelihood of substance use. That is, members of a specific social scene create symbolic boundaries and develop tastes around a music genre and given substances, reinforcing that they are complementary and scene appropriate (Vuolo et al., 2014, Kelly et al., 2015, Cournoyer Lemaire et al., 2021). A considerable amount of research in nightlife settings focuses on electronic dance music (EDM), as studies reveal that drug use is particularly prevalent among people who attend EDM parties at nightclubs or festivals (Palamar, Lee, et al., 2023; van Dyck et al., 2023), though some studies also include other genres of music, such as hip-hop, rap, and rock (e.g., Erbella et al., 2020; Pawson and Kelly, 2014; Rig & Estreet, 2018; van Havere et al., 2011, van Havere et al., 2012). Furthermore, research consistently shows that drug use in nightlife venues is higher among LGBTQ+  populations compared to heterosexual individuals (Lea et al., 2013, Hunt et al., 2019), and is sometimes used to enhance sexual experiences – a practice known as ‘chemsex’ (Íncera-Fernández et al., 2021, Santoro et al., 2020). While nightlife patrons are a subset of the population overall, *in situ* studies of nightlife also provide the ability to enter these subcultures and scenes.

In nightlife settings, a wide variety of substances are commonly used, including licit drugs such as alcohol and tobacco, traditional illicit drugs such as cannabis, MDMA, and cocaine, prescription medications used non-medically, and new psychoactive substances (NPS) (European Monitoring Centre for Drugs and Drug Addiction, 2018, Kelly and Vuolo, 2020, Nordfjærn et al., 2016, Palamar and Le, 2023), and those using them are not homogeneous group (Hannemann et al., 2017, Santos et al., 2015). Polydrug use, specifically the use of multiple substances simultaneously, is common in nightlife environments and especially raises concerns as it amplifies the effects of individual drugs, and, combined with lack of awareness of its harmful effects, may lead to harmful physiological damage and increased risk of physical harm (Feltmann et al., 2021; Kirtadze et al., 2022; Quek et al., 2013). Polydrug use is strongly correlated with both the frequency of nightclub attendance and the nightclub's musical style (Sañudo et al., 2015). Techniques only possible *in situ*, such as the collection of biomarkers alongside surveys, permit the ability to gather real-time information on substances being used.

Acute intoxication from certain substances is associated with heightened risks; however, they vary according to the type of substance and individual-level characteristics. Adolescents and young adults are at higher risk for binge drinking and experimentation with illicit substances, and related harms such as violence, injuries, risky sexual behavior, and impaired driving (e.g. Calafat et al., 2009; Pedersen et al., 2016). Older individuals, who are less likely to engage in high-frequency nightlife substance use, may face heightened health consequences from alcohol or polydrug use due to age-related comorbidities (Rehm, 2011). Men are more likely to engage in heavy episodic drinking and polydrug use in nightlife settings, and are more often involved in substance-related violence (Nordfjærn, 2017, Jørgenrud et al., 2021), while women, who generally consume less alcohol and drugs, may experience greater acute physiological effects at equivalent alcohol doses due to body composition and hormonal differences (Thomasson, 2002, Vergeer et al., 2025) and are at higher risk of experiencing sexual violence when intoxicated (Fileborn, 2012). Moreover, prolonged substance use may be associated with mental health issues (Akunna et al., 2024, Keaney et al., 2011), such as impairments in mood, sleep and memory functioning (Feltmann et al., 2021), but also physical manifestations like palpitations, respiratory depression, and collapsing (Feltmann et al., 2021; Mirijello et al., 2023). Additionally, substance use is also related to reduced productivity (Frone, 2004), and disruptions in educational or work activities (Lehman and Bennett, 2002, Mekonen et al., 2017). Pre-existing mental health conditions (e.g., anxiety, depression) may increase vulnerability to substance misuse and adverse outcomes (Liang et al., 2011). All these issues highlight that substance use in nightlife settings is a significant public health concern, which requires a variety of methods to gather data that can help address these concerns.

In sum, previous research on nightlife behaviors reveals key patterns in substance misuse, as well as various risk factors which may be associated with negative consequences. Gaining insight into these factors is important for understanding the complexities of nightlife settings and designing effective interventions to minimize substance-related harm among nightlife patrons. We focused on *in situ* studies in order to review what information can be obtained and what types of results can be concluded from this form of on-site data collection. For example, it offers unique opportunities to collect both survey and biological data about real-time substance use in an environment where substance use is common, but also has potential challenges such as participant selection, issues of intoxication, and the need for quick data collection given the interruption to participants’ socializing. Although considerable research has been conducted *in situ*, to our knowledge, no systematic review has yet been carried out on this topic. Therefore, the aim of this systematic review is to explore empirical *in situ* research in nightlife settings, with focus on substance use.

## 2 Methods

The systematic literature search was conducted in the Web of Science (WOS), PsycInfo, PubMed, and Google Scholar databases. The following search term was used: nightlife OR nightlife setting OR nightclub OR nighttime entertainment OR music festival OR recreational setting(s) AND risk behaviors OR psychoactive substances OR alcohol OR binge drinking OR heavy episodic drinking OR intoxication OR illicit drugs OR recreational drugs OR psychostimulants OR stimulant type drugs OR MDMA OR ecstasy OR amphetamines OR methamphetamines OR cannabis OR cocaine OR new psychoactive substances OR ketamine OR GHB/GBL OR nitrous oxide OR synthetic cannabis OR synthetic stimulants OR synthetic cathinones OR medications OR prescription drugs OR polysubstance use. In WOS “topic” (title, keywords, abstract) was searched, in PubMed “title/abstract”, in Google Scholar “default search”, and in PsycInfo “title” and “keywords”. Inclusion criteria were: (1) articles published between January 2014 until December 2023; with data collected after 2010; (2) published in English; (3) empirical research conducted *in situ* in nightlife settings; (4) substance use as an outcome. Exclusion criteria were: (1) articles not including empirical data (e.g., theoretical articles, policy articles, research protocols); (2) grey literature (e.g., dissertation, master paper, reports); (3) articles including only data on tobacco use, (4) outcome not related to substance use, (5) articles including data collected *in situ* at music festivals only, (6) research not conducted *in situ* in nightlife settings. We do not consider surveys not collected *in situ* that may have asked about nightlife participation nor those that solely recruited or screened participants *in situ* for later data collection in another location or online, as they do not share most of the same opportunities and challenges. Focusing on *in situ* data collection only still returned a substantial number of articles.

The preselection process involved reviewing the title and abstract of each study. The full texts of the preselected studies were then accessed and assessed for eligibility based on the inclusion criteria. Any discrepancies in selection and coding were resolved through discussions among all authors.

In the first phase of the search protocol, a total of 1410 articles were identified. The WOS database contained 610 articles, PsychInfo 316, PubMed 384, and Google Scholar 100 articles (top 100 results). Before screening the articles, 702 duplicate records were removed. The 708 remaining articles were then screened by title and abstracts, resulting in the exclusion of 533 articles not relevant to the present review, leaving a total of 175 articles eligible for further review. In the second phase, a full-text analysis and consultation between the authors were conducted. We excluded 120 studies because they were either not conducted *in situ*, did not focus on substance use as an outcome (e.g., assessed quality of biospecimens. preferred bar characteristics, hospital admissions), were published before 2014, or reported on research conducted before 2010. Altogether, 55 articles were included in this review.

**Fig. 1:**
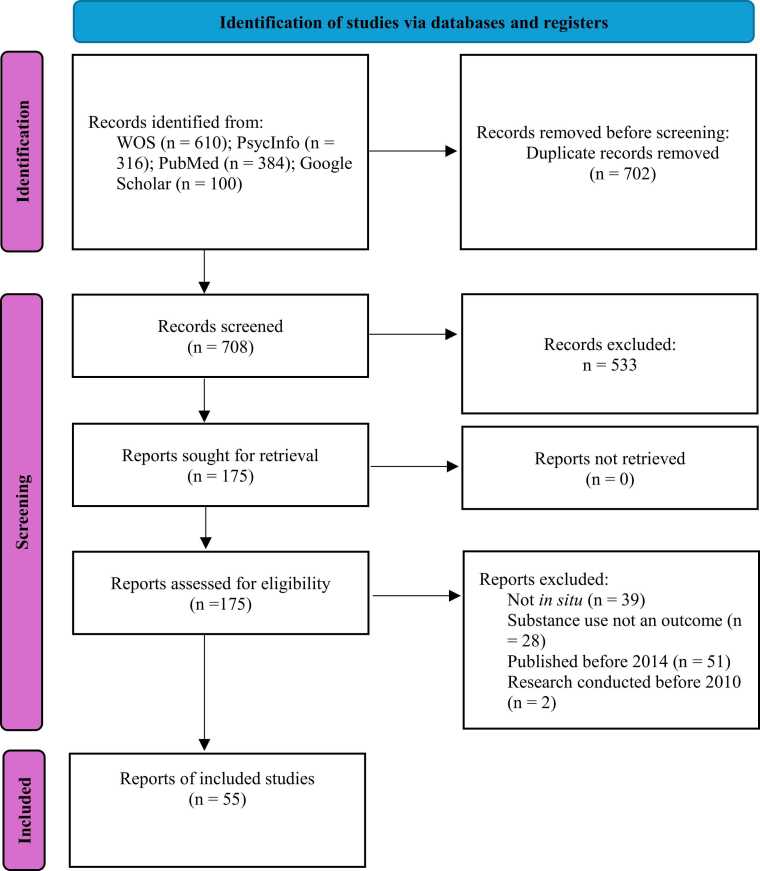
PRISMA Flow Diagram.

The search process followed the guidelines outlined in the Preferred Reporting Items for Systematic Reviews and Meta-Analyses (PRISMA) statement (Page et al., 2021) and is presented in the PRISMA flow diagram (see Fig. 1).

## 3 Results

### 3.1 Geographic location

Table 1 reports the characteristics of the 55 studies included. Among these 55 articles, many came from the same larger study. For example, 13 articles were from the same long-term study of nightlife in New York City largely focusing on the EDM scene (24 %; e.g., Palamar, 2023; Palamar, Salomone et al., 2023), nine were from a five-city study in Australia using street interception in nightlife districts (16 %; e.g., Miller, Curtis, Jenkinson et al., 2015), and nine were from related studies of nightlife venues in São Paulo (16 %; e.g., Santos, Paes, et al., 2015). By contrast, there was virtually no repetition from studies from Europe (with the exception of two articles from the same study of Copenhagen; Bøhling, 2014, Bøhling, 2015), representing a wide variety of countries. No other world region was represented, totaling 35 % from the U.S. (27 % in New York City), 24 % European countries, 25 % Australia or New Zealand, and 16 % Brazil (all São Paulo).

**Table 1:** A summary of the methodology and main findings of the reviewed articles.

**Authors**	**Data collection year**	**Country (City)**	**Collection techniques**	**Bio-marker source**	**Analytic N**	**Venue type**	**Method of participant selection**	**In situ participation rate**	**Compen-sation**	**% male**	**Age as reported**	**% hetero**	**Main findings**
Baldin et al. (2018)	2013	Brazil (São Paulo)	survey	breath	465	any with dance floor	every third entrant	79 %		64.5 %	24.7 (6.0); R: 18–55		Modest effects of a web-based intervention within-group for binge drinking, but not between-group to a control.
Bøhling (2014)	N/A	Denmark (Copenhagen)	ethnography, in-depth interviews		15	nightlife district, EDM							People, drugs, music, and space converge to generate emotion.
Bøhling (2015)	N/A	Denmark (Copenhagen)	ethnography, in-depth interviews		18	one dance-pop nightclub							Perceptions of drunkenness also associated with time and space.
Bourdeau et al. (2015)	2010–12	USA (San Francisco)	survey, biomarker	breath, oral	1833	EDM nightclubs	portal method - first to cross imaginary line near entrance	60 % approached stopped; 63 % participated	$10 enter, $20 exit	51.4 %	27.7 (7.60)	77.6 % sample 1; 59.7 % sample 2	Taking alternative form of transportation associated with higher BAC.
Bretteville-Jensen et al. (2019)	2014	Norway (Oslo)	survey, biomarker	breath, oral	1085	nightclubs		76 % consent rate	Free food	63.7 %	26.9 (6.8)		Positive test more likely among current using groups, but also detected in never and previous use groups.
Byrnes, Miller, Bourdeau, Johnson (2019)	2015	USA (San Francisco)	survey, biomarker	breath, oral	815	EDM nightclubs	portal method - first to cross imaginary line near entrance	59 % of those who stopped	$15 enter, $25 exit	55.8 %	27.7 (6.0)		Group cohesion is associated with having strategies to keep group safe.
Byrnes, Miller, Bourdeau, Johnson, et al. (2019)	2016–17	USA (San Francisco)	survey, biomarker	breath, oral	959	EDM nightclubs	portal method - first to cross imaginary line near entrance	25 % of groups who stopped	$15 enter, $25 exit	54.7 %	28.0 (6.35)		Intervention participants used more protective actions to keep group members safe, had lower BACs.
Cameron et al. (2018)	2014	New Zealand (Hamilton)	survey, biomarker	breath	320	nightlife district	every seventh pedestrian			67.2 %	28.2 (11.6); R: 18–70		Women’s intoxication levels stop increasing after midnight; men’s increase through the night; intoxication remains higher in individuals who engage in pre-drinking than among those who do not.
Carlini et al. (2014)	2013	Brazil (São Paulo)	survey, biomarker, environmental	breath	1822	nightclubs	every third entrant	80 % entrance, 76 % follow up		61.0 %	R: 18–66		Pre-drinking strongest predictor of exit BAC, alongside number of dance floors, televisions, sound level, and ‘all you can drink’ service.
Carlini et al. (2017)	2013	Brazil (São Paulo)	survey, biomarker, environmental	breath	1822	nightclubs	every third entrant			60.7 %	25.0 (0.91)		Licit and illicit drug use positively associated with all-you-can-drink service, as well as dance floors and bouncers
Carvalho et al. (2019)	2017	Portugal (Porto)	survey, biomarker	breath	830	nightlife district	no pattern: those interested in knowing their BAC	21 %		71.3 %	30.6 (9.2); R: 18–67		High BAC individuals underestimate BAC results, opposite for low BAC; high BAC individuals report will affect later decisions, such as driving
Chan et al. (2016)	2013	UK (London)	survey		313	MSM nightclubs	no pattern: attendees			90.1 %	31.3 (7.6); R: 18–60	20.8 %	Reported prevalence of prescription and OTC medications
Chaney et al. (2019)	2015	USA (unspecified southeastern city)	survey, biomarker	breath	548	college bar district	no pattern: attendees			62.2 %	21.6 (3.5); R: 18–48		Predrinking explains most variance in BAC and is linked to hazardous drinking behavior.
Coomber et al. (2018)	2016	Australia (Brisbane)	survey, biomarker	breath	1059	nightlife district	every third passee	84 %		65.0 %	23.0 (5.9)		Post-intervention, more people went out earlier and a decrease was observed in high BAC
Crocamo et al. (2018)	N/A	Italy (Milan)	survey		507	pubs, discos, or live music events				48.0 %			D-ARIANNA model showed an appreciable, though modest, predictive ability for subsequent BD episodes.
Curtis et al. (2017)	2011–12	Australia (Sydney, Melbourne, Perth, Geelong, Wollongong)	survey, biomarker	breath	4203	nightlife district	every third person			59.0 %	25.0 (6.94)		Intention to consume alcohol and drugs associated with being male and high BAC.
Devilly et al. (2021)	2015–19	Australia (Brisbane)	survey, biomarker	breath	2670	nightlife district	no pattern: those outside bars and in nightlife district			pre: 58.30 %; post: 59.71 %	pre: 24.38 (6.43); post: 24.68 (7.26)		Legislation did not affect BAC among those in nightlife area
Droste et al. (2016)	2011–12	Australia (Sydney, Melbourne, Perth, Geelong, Wollongong)	research survey of environment			pubs, bars, nightclubs catering to different scenes							Alcohol mixed with energy drinks positively associated with later time, high intoxication levels, illicit drug use, and younger crowds
Droste et al. (2018)	2011–12	Australia (Sydney, Melbourne, Perth, Geelong, Wollongong)	survey, biomarker, interviewer ratings	breath	5273	nightlife district	every third person	93 %		61.5 %	23.9 (6.36); R: 18–73		As BAC increases, visible intoxication symptoms increase as well as interview-participant rating disagreement
Erbella et al. (2020)	2013	Brazil (São Paulo)	survey, biomarker	breath	369	nightclubs	no pattern: attendees						Signs of altered psychomotor capacity among drivers associated with BAC
Gilmore et al. (2022)	2015–16	Australia (Perth)	survey, biomarker	breath	667	nightlife district	every third person	89 %		68.1 %	19–21 = 19.0 %; 22–25 = 28.5 %; 26–29 = 23.3 %; > =30 = 29.3 %		AUDIT-C associated with higher BAC, moderated for males by preferred bar type
Griffin et al. (2019)	2016–18	USA (New York)	survey		3066	nightclub parties	time-space venue sampling, then all entrants	75 %	$10	56.1 %	18–24 = 59.8 %; 25–40 = 40.2 %	82.4 %	Specific drug use associations differ by sexual identity
Hannemann et al. (2017)	N/A	Germany (Munich)	survey		1571	EDM events	no pattern: those approaching a booth			61.6 %	23.1 (4.7)		4 classes of individuals who use NPS : cannabis limited; traditional club drugs; club and psychedelics; non-selective/all drugs
Hughes et al. (2019)	2014–15	UK (3 unspecified)	survey		408	nightlife district	no pattern: passersby	City 1: 50.6 %; City 2: 48.7 %; City 3: 57.2 %		55.6 %	18–20 = 33.3 %; 21–24 = 34.6 %; 25–35 = 32.1 %		Higher expected drunkenness associated with higher ideal drunkenness, higher perceived drunkenness norm, and later expected home time
Hughes et al. (2023)	2014–17	Australia (Queensland)	survey, biomarker	breath	4723	nightlife district	every fourth passerby						Preloaders younger, male, intoxicated upon arrival, and affected subjectively by BAC; more admit to using drugs when police not present
Jørgenrud et al. (2021)	2017	Norway (Oslo and 6 small cities within 2 h)	survey, biomarker	breath, oral	1988	dance music venues (EDM or pop)	no pattern: attendees		voucher for free food	62.0 %	16–20 = 15.2 %; 21–25 = 45.8 %; 26–35 = 27.2 %; 36 + = 10.8 %		Provides drug prevalence estimates
Kaestle et al. (2018)	2011–12	Australia (Sydney, Melbourne, Perth, Geelong, Wollongong)	survey, biomarker	breath	4628	nightlife district	every third person			64.3 %	under 21 = 34.3 %; over 21 = 63.7 %		Staying out late, pre-drinking, and being young biased toward higher self-assessed intoxication regardless of BAC. Energy drinks and stimulant used decreased ability to perceive BAC differences
Kelly et al. (2014)	2011–13	USA (New York)	survey		1526	bars, clubs, lounges, performance venues	time-space venue sampling, then all entrants	78 %		52.5 %	24.3 (2.67)	64.2 %	Prescription drug misuse is combined with several other drugs, associated with gender and sexual identity.
Mandler (2016)	2015	USA (New York)	ethnography, in-depth interviews		18	queer bars, clubs, warehouses, after-hours venues	purposive qualitative sampling of venues			inten-tionally does not classify		0.0 %	Chemical use enables queer nightlife workers perform and produce pleasure for others, combining functional and pleasurable use, and have their own harm reduction strategies
Miller, Curtis, Jenkinson et al. (2015)	2011–12	Australia (Sydney, Melbourne, Perth, Geelong, Wollongong)	survey, biomarker	oral	7028	nightlife district	every third person (survey); every fifth person (biomarker)			61.2 %	median: 22; R: 18–73		Psychostimulants most commonly used in both saliva and self-report; good agreement in measures.
Miller, Droste, Martino et al. (2015)	2010–11	Australia (Newcastle, Geelong)	survey		3949	nightlife venues	every third person			54.4 %	24.3 (5.8)		Those consuming drugs more likely have prior violent incident, higher self-reported intoxication, and high risk alcohol use
Miller et al. (2014)	2011–12	Australia (Sydney, Melbourne, Perth, Geelong, Wollongong)	survey, biomarker	breath	6998	nightlife district	every third person	97 %		61.8 %	24.89 (6.37); R: 18–73		BAC increases throughout the night, with differences by sex disappearing and age differences emerging
Nordfjærn et al. (2016)	2014	Norway (Oslo)	survey, biomarker	breath	1099	licensed nightlife venues	no pattern: attendees	76 %		64.3 %	27.0 (6.78); R: 16–64		Provides drug prevalence estimates, with highest rates among teens, males, and those initiating younger
Palamar and Le (2022)	2019–20	USA (New York)	survey		209	EDM venues	time-space venue sampling, then all entrants	76 %		59.5 %	18–24 = 30.2 %; 25–29 = 43.2 %; ≥ 30 = 26.2 %	76.2 %	Ketamine use generally not associated with media coverage, but possible among some subgroups.
Palamar and Le (2023)	2019–22	USA (New York)	survey		1952	EDM venues	time-space venue sampling, then all entrants	2019: 65 %; early 2020: 82 %; 2021: 63 %; 2022: 82 %	$10	55.0 %	26.7 (6.0)	67.7 %	Adverse effects higher for less prevalent drugs and younger age and lower with greater number of drugs used; taking too much most common reason, hospital visit most common outcome.
Palamar and Salomone (2023)	2019–22	USA (New York)	survey, biomarker	hair	328	EDM venues	time-space venue sampling, then all entrants	69 %	$15	52.4 %	26.1 (5.9)	69.8 %	Underreporting of drug use was common, but could be due to adulterants
Palamar (2023)	2018–22	USA (New York)	survey		2981	EDM venues	time-space venue sampling, then all entrants	73 %	$10	57.4 %	18–25 = 48.3 %; > =26 = 51.7 %	73.8 %	Personally experienced adverse effects can deter willingness to use certain party drugs again
Palamar, Acosta, and Cleland (2019)	2017	USA (New York)	survey		954	EDM venues	no pattern: attendees	74 %	$10 enter, $20 gift card follow-up	51.6 %	25.4; R: 18–40	83.5 %	Unplanned drug use common at EDM parties, more common at nightclubs than festivals
Palamar, Acosta, Calderón et al. (2017)	2016	USA (New York)	survey		1048	EDM venues	no pattern: attendees	77 %	$10	57.4 %	R: 18–40		Reported prevalence of some NPS higher with "gate question" on overall NPS use.
Palamar, Acosta, Le et al. (2019)	2018	USA (New York)	survey		1029	EDM venues	time-space venue sampling, then all entrants	73 %	$10	58.7 %	18–24 = 49.5 %; 25–40 = 50.5 %	81.5 %	Adverse effects from drug use common among those in the EDM party scene, and polydrug use a common risk factor
Palamar, Acosta, Ompad et al. (2017)	2015	USA (New York)	survey		679	EDM venues	time-space venue sampling, then all entrants	63 %	$10	54.6 %	21.9 (0.2); R:18–25	83.1 %	Ecstasy use common, especially among older, frequent attendees and higher peer exposure; those using ecstasy more likely to report other drugs use
Palamar, Acosta, Sherman et al. (2016)	2015	USA (New York)	survey		682	EDM venues	time-space venue sampling, then all entrants	63 %	$10	54.7 %	18–20 = 26.1 %, 21–22 = 33.3 %; 23–25 = 40.5 %	83.2 %	Risk factors for lifetime NPS use included MDMA, LSD, and ketamine use, identifying as bisexual, more frequent attendance, particularly festivals
Palamar, Le, Cleland et al. (2023)	2017–2022	USA (New York)	survey		1029	EDM venues	time-space venue sampling, then all entrants	2017: 74 %, 2018: 73 %, 2019: 65 %, 2021: 63 %, 2022: 82 %	$10	55.8 %	< 26 = 46.2 %; ≥ 26 = 53.8 %	79.1 %	From 2017–2022, increases in past-year use of poppers, ketamine, and shrooms; past-month use of 2 C series and novel psychedelics; decreases in use of ecstasy, cocaine, and prescription opioids.
Palamar, Salomone, Massano et al. (2023)	2016–22	USA (New York)	survey, biomarker	hair	247	EDM venues	time-space venue sampling, then all entrants	72 %	$10	53.0 %	18–25 = 51.0 %; > =26 = 49.0 %		Combined self-report and toxicology tests and found decreases in drug use/exposure were steeper; underreported drug exposure decreased over time.
Palamar, Salomone, Vincenti et al. (2016)	2015	USA (New York)	survey, biomarker	hair	48	EDM venues	time-space venue sampling, then all entrants	survey: 63 %; biomarker: 26 %	$10	full: 54.6 %; hair: 64.6 %	full: R: 18–25; hair: 18–20 = 20.8 %, 21–22 = 39.6 %, 23–25 = 39.6 %		Many ecstasy-using nightclub/festival attendees may be unintentionally using “bath salts” or other NPS
Peacock et al. (2016)	2011–12	Australia (Sydney, Melbourne, Perth, Geelong, Wollongong)	survey, biomarker	oral	5556	nightlife district	every third person	93 %		62.0 %	median: 22; R: 20–26		One-third of individuals who use alcohol have low risk consumption practices; elevated harms amongst those pre-drinking and using illicit drugs.
Pennay et al. (2016)	2011–12	Australia (Sydney, Melbourne, Perth, Geelong, Wollongong)	survey, biomarker	oral	6984	nightlife district	every third person	93 %		61.9 %	median: 22; R: 18–73		Harms, such as intoxication-related accidents/injuries, particularly reported by those using cannabis and cocaine.
Sanchez et al. (2015)	2013	Brazil (São Paulo)	survey, biomarker	breath	1222	nightclubs and discos	random venue sample, then every third entrant	79 %		56.8 %	18–24 = 51.1 %; 25–34 = 35.8 %; 35–44 = 10.5 %; 45 + = 2.6 %		The most prevalent risk behaviors practiced after leaving a nightclub were drinking and driving, the use of illicit drugs and risky sexual behavior.
Santos, Baldin, Carlini et al. (2015)	2013	Brazil (São Paulo)	survey, biomarker	breath	1057	nightclubs and discos	random venue sample, then every third entrant			53.6 %	18–24 = 50.4 %; 25–34 = 36.6 %; 35–44 = 10.3 %; 45 + = 2.7 %		Half of the nightclub patrons presented any alcohol use disorder, associated with being male, lower education, younger, increased illicit drug use.
Sañudo et al. (2015)	2013	Brazil (São Paulo)	survey, biomarker	breath	2422	nightclubs and discos	random venue sample, then every third entrant	79 %		60.7 %			Predrinking more prevalent among men; however, men and women who engaged in predrinking have a similar alcohol consumption. Predrinking associated with male intoxicated driving and female experience of sexual harassment.
Sañudo et al. (2015)	2013	Brazil (São Paulo)	survey, biomarker	breath	2420	nightclubs and discos	random venue sample, then every third entrant	79 %		60.8 %	18–25 = 62.9 %; 26–33 = 24.9 %; > =34 = 12.2 %		Polydrug use more likely among men, young adults, and frequent attendees.
Sañudo et al. (2018)	2013	Brazil (São Paulo)	survey		2422	nightclubs and discos	random venue sample, then every third entrant	79 %		60.8 %	18–25 = 62.9 %; 26–33 = 24.9 %; > =34 = 12.2 %		Risky alcohol use higher among males, high SES, and those at hip-hop venues.
Thurtle et al. (2017)	2015	UK (London)	survey		101	One gay nightclub	no pattern: those coming to "chill out" zones			89.0 %	median: 28; IQR: 23–34	19.0 %	5.9 % of participants reported using ENDS to vape substances other than nicotine.
Waldron et al. (2020)	2017	Belgium, Italy, Netherlands, UK	survey		3529	nightclubs	random intercept method: every second person in imaginary zone	76 %		58.0 %	24.4		Offline sample used drugs and attended venues more frequently, but small effect sizes imply online recruitment accesses similar population as random sampling
Wieczorek et al. (2021)	2016	Poland (Warsaw, Krakow, Poznan, TriCity)	survey		172	event venues	no pattern: recruitment by party workers and snowball			66.7 %	24.8; 18–24 = 56.1 %; 25–34 = 39.8 %; > =35 = 4.1 %		Stimulant/empathogen/ nootropics group were the most commonly used among nightlife recruited group; also higher mental health conditions.

### 3.2 Data collection techniques

One of the main benefits of *in situ* data collection appeared to be the ability to obtain biomarker data. This information was then used for a variety of purposes: as both an independent and dependent variable, to compare to survey responses of the same measure, and to use this comparison to deduce drug quality such as adulteration. Thirty-one studies (56 %) collected such information. Across all studies, 36 % collected biomarkers using breath only, 5 % using oral only, 9 % using both breath and oral, and 5 % using hair. In every instance where biomarker data was collected, the researchers also administered a survey. Survey data collection was by far the most common technique among all articles, with 51 of the 55 articles doing so (93 %). Two survey studies (that were among those collecting biomarkers) also had researchers code for environmental factors (Carlini et al., 2014, Carlini et al., 2017), while one study’s sole data collection technique was such environmental factors (Droste et al., 2016). The remaining three articles constituted ethnographic and/or in-depth interview data collection methods (Bøhling, 2014, Bøhling, 2015, Mandler, 2016).

### 3.3 Other study design considerations

The articles reported several different techniques for participant selection, which can be categorized similarly. First, 17 studies (31 %) used “every X person” as the sole technique. However, every person from what varied, with studies alternatively reporting entrants or attendees (Carlini et al., 2014, Carlini et al., 2017, Baldin et al., 2018), pedestrians or passersby (Cameron et al., 2018, Hughes et al., 2023), those who enter a zone with an imaginary border (“random intercept method”; Waldron et al., 2020), those crossing an imaginary line (“portal method”; Bourdeau et al., 2015; Byrnes, Miller, Bourdeau, and Johnson, 2019; Byrnes, Miller, Bourdeau, Johnson et al., 2019), or generically “person” (Curtis et al., 2017, Droste et al., 2018, Gilmore et al., 2022, Kaestle et al., 2018, Miller et al., 2015, Miller et al., 2015, Miller et al., 2014, Peacock et al., 2016, Pennay et al., 2016). Second, five articles all from the same São Paulo study (9 %) reported combining random venue selection with “every X person” (e.g., Santos, Paes, et al., 2015). Third, the 12 aforementioned articles from the same study of New York City (29 %) selected all attendees after randomly selecting venues and time, known as time-space sampling (e.g., Palamar, 2023; Palamar, Salomone, et al., 2023). Fourth, some described non-random methods. (We note that in some instances, the method could potentially be inferred from other articles from the same study; however, we coded the articles based on their self-contained content.) While purposive and snowball sampling are typical and accepted techniques for the three included qualitative studies (Bøhling, 2014, Bøhling, 2015, Mandler, 2016), there were also non-random survey studies. Such reported techniques included those coming to an information booth or a chill-out zone or booth (Thurtle et al., 2017, Hannemann et al., 2017), snowball sampling (Wieczorek et al., 2021), or what appeared to otherwise be a convenience sample (Carvalho et al., 2019, Chan et al., 2016, Chaney et al., 2019, Devilly, 2021, Erbella et al., 2020, Hughes et al., 2019, Jørgenrud et al., 2021, Nordfjærn et al., 2016, Palamar et al., 2019, Palamar et al., 2017). Finally, we note that although we concentrate on data collection *in situ* in nightlife and this reflects the information reported in Table 1, we note that some studies included other surveys as complements to the *in situ* collection (e.g., Waldron et al., 2020; Wieczorek et al., 2021) and several included additional data collection upon leaving a venue (e.g., Bourdeau et al., 2015; Byrnes, Miller, Bourdeau, and Johnson, 2019; Byrnes, Miller, Bourdeau, Johnson, et al., 2019; Carlini et al., 2014).

Regarding what spaces respondents were located in when being sampled, the three main locations were those in a nightlife district, those entering specific venues, and those already inside a venue. Those using a district as the location were mostly unspecified beyond where nightlife venues were located (16 articles: Bøhling, 2014; Cameron et al., 2018; Carvalho et al., 2019; Chaney et al., 2019; Coomber et al., 2018; Curtis et al., 2017; Devilly, 2022; Droste et al., 2018; Gilmore et al., 2022; Hughes et al., 2019, Hughes et al., 2023; Kaestle et al., 2018; Miller, Curtis, et al., 2015; Miller et al., 2014; Peacock et al., 2016; Pennay et al., 2017). When venues were specified, many articles described them in generic terms such as nightclubs, clubs, discos, or events (e.g., Bretteville-Jensen et al., 2019; Carlini et al., 2014, Carlini et al., 2017; Crocamo et al., 2018; Droste et al., 2016; Erbella et al., 2020; Griffin et al., 2020; Kelly et al., 2014; Sanchez et al., 2015; Santos, Paes, et al., 2015; Waldron et al., 2020). Among specified venue types, the most common were EDM venues and/or other “dance” venues (20 articles: Baldin et al., 2018; Bøhling, 2014, Bøhling, 2015; Bourdeau et al., 2015; Byrnes, Miller, Bourdeau, and Johnson, 2019; Byrnes, Miller, Bourdeau, Johnson, et al., 2019; Hannemann et al., 2017; Jørgenrud et al., 2021; Palamar and Le, 2022, Palamar and Le, 2023; Palamar and Salomone, 2023; Palamar, 2023; Palamar, Acosta, and Cleland, 2019; Palamar, Acosta, Calderón, et al., 2017; Palamar, Acosta, Le, et al., 2019; Palamar, Acosta, Ompad, et al., 2017; Palamar, Acosta, Sherman, et al., 2016; Palamar, Le, Cleland, et al., 2023; Palamar, Salomone, et al., 2023; Palamar, Salomone, et al., 2016). Three articles specifically collected data only at venues known to be frequented by sexual minority people; that is, the gay or queer scene (Thurtle et al., 2017, Mandler, 2016, Chan et al., 2016).

Excluding the three qualitative studies and one survey article not reporting it, the *in situ* sample size varied dramatically across articles. The average was 1953.2, ranging from 48 (Palamar, Salomone, et al., 2016) to 7028 (Miller, Curtis, et al., 2015). We note, however, that the reported analytic sample size was occasionally a subset of the total sample due to the subset being examined (e.g., only those with biomarker data, reporting use of a specific substance, or data collected in particular years). For recruitment, 31 % of articles reported some form of compensation, either a small amount of money or a voucher for free food (Bourdeau et al., 2015, Bretteville-Jensen et al., 2019, Byrnes et al., 2019, Byrnes et al., 2019, Griffin et al., 2019, Jørgenrud et al., 2021, Palamar and Le, 2023, Palamar and Salomone, 2023, Palamar, 2023, Palamar et al., 2019, Palamar et al., 2017, Palamar et al., 2019, Palamar et al., 2017, Palamar et al., 2016, Palamar et al., 2023, Palamar et al., 2023, Palamar et al., 2016). Perhaps the hardest characteristic to easily summarize is the information on the rate of participation. We were surprised not to find any such information in 21 articles (Bøhling, 2014, Bøhling, 2015, Cameron et al., 2018, Carlini et al., 2017, Chan et al., 2016, Chaney et al., 2019, Crocamo et al., 2018, Curtis et al., 2017; Devilly et al., 2021; Droste et al., 2016; Erbella et al., 2020; Hannemann et al., 2017; Hughes et al., 2023; Jørgenrud et al., 2021; Kaestle et al., 2018; Mandler, 2016; Miller, Curtis, et al., 2015; Miller, Droste, et al., 2015; Santos, Baldin, et al., 2015; Thurtle et al., 2017; Wieczorek et al., 2021). Even among those reporting such rates, they differ on the precise information included, such whether the consent rate or participation rate is included, percentage eligible of those screened, and whether differences exist between the percentage who were willing to stop versus subsequently agreeing to participate. Typical response rates tended to be in the range of 60–80 %, although several studies reported a high of 93 %.

### 3.4 Demographic characteristics

Given what is known about nightlife participation, the demographics (when reported) were as might be expected. Every study reported a majority male. There was a variety of ways the articles reported age. However, the result is consistent that the participants are overwhelmingly young adults. For those studies reporting a mean or median, all noted a mean in the twenties, except two that reported means just over thirty (30.6 and 31.3). For those studies that reported age categories, making this determination is more difficult given inconsistent age cutoffs, but young adults were still typically the most common group. Only 15 studies (27 %) reported on sexual orientation (Bourdeau et al., 2015, Chan et al., 2016, Griffin et al., 2019, Kelly et al., 2014, Mandler, 2016, Palamar and Le, 2022, Palamar and Le, 2023, Palamar and Salomone, 2023, Palamar, 2023, Palamar et al., 2019, Palamar et al., 2019, Palamar et al., 2017, Palamar et al., 2016, Palamar et al., 2023, Thurtle et al., 2017). While heterosexual tends to be the most common category, sexual minority people were still a large proportion, often as a result of study design considerations that sample nightlife scenes frequented by sexual minority people. Studies used a variety of categorizations for sexual minority groups, such that we do not display them in the table, but they typically included gay, lesbian, bisexual, and an “other” group. Few studies reported on transgender identity (e.g., Kelly et al., 2014), or specifically stated it was inappropriate to categorize by sex or gender given the scene under study (Mandler, 2016).

### 3.5 Substances reported

Table 2 shows the substances mentioned across the articles. We coded for any mention that the information on the substance was collected, even if it was not the main focus of the article. While undoubtedly many surveys described in articles included other substances in their data collection (as can be deduced from articles from the same study), we only describe here substances explicitly mentioned within an article. Further, both here and in the next section, we note that while many studies assessed use at the time of the survey, several also contained retrospective self-reports.

**Table 2:** Frequency of substances mentioned across included articles.

Substances	Percentage of articles (%)
**Licit substances and pharmaceuticals**	
Alcohol	76.4
Tranquilizers/benzodiazepines	32.7
Other prescription and over-the-counter medicines	23.6
Tobacco	18.2
Energy drink consumption	14.5
E-cigarettes	3.6
**Illicit drugs**	
Illicit drugs (general)	30.9
MDMA/Ecstasy	60.0
Cocaine	58.2
Cannabis	56.4
Ketamine	54.5
Amphetamines	45.5
Methamphetamines	41.8
LSD	36.4
Heroin	30.9
GHB	27.3
Mushrooms	16.4
Psilocybin	14.5
Hallucinogens (general)	14.5
Inhalants (e.g., nitrous; poppers)	14.5
Crack	12.7
DMT	7.3
**Novel Psychoactive Substances**	34.5

Beginning with licit drugs and pharmaceuticals, alcohol was the most common substance reported collected at 76.4 % of articles. Notably, 12.7 % of articles collected information on use of alcohol prior to arrival to the nightlife environment, alternatively known as pre-drinking, pre-loading, or pre-gaming, depending on the geographic context (Cameron et al., 2018, Carlini et al., 2014, Chaney et al., 2019, Hughes et al., 2023, Kaestle et al., 2018, Peacock et al., 2016, Santos et al., 2015). For nicotine, 18.2 % of articles collected information on tobacco (including one on snus/snuff) and 3.6 % information on e-cigarettes (including one examining vaping illicit substances as well). We reiterate that we did not include articles whose sole focus was tobacco/nicotine, such that these numbers should be considered as also being collected alongside other licit and illicit substances. Similarly, energy drink consumption articles were not included on their own, but 14.5 % of the articles in our review did collect information on energy drinks, typically in combination with drinking alcohol. Finally, articles commonly collected information on pharmaceuticals, with 32.7 % doing so for tranquilizers/benzodiazepines and 23.6 % collecting data on other prescription or over-the-counter medicine. We note that it was not always possible to determine if prescription drug use could be considered misuse as opposed to for medical purposes.

Illicit drugs were commonly collected as well. A total of 30.9 % of the articles did not distinguish, but instead included a general collapsed illicit drug variable. Where distinguished, 56.4 % of articles collected information on cannabis, 30.9 % heroin, and 58.2 % on cocaine with another 12.7 % crack cocaine. Given their use in nightlife, a high rate of articles collected data on MDMA/ecstasy (60.0 %), ketamine (54.5 %), and GHB (27.3 %) as well as stimulants in the form of amphetamines (45.5 %) and methamphetamine (41.8 %). A general hallucinogen was mentioned in 14.5 % of articles, but more specific substances were also collected: 36.4 % LSD, 16.4 % mushrooms, 14.5 % psilocybin, and 7.3 % DMT. Inhalants, typically either nitrous oxide or poppers, were collected in 14.5 % of articles. Finally, a very heterogeneous group of Novel Psychoactive Substances were collected in 34.5 % of articles.

### 3.6 Narrative of main findings

The articles included in the systematic review are quite varied in their focus. In order to provide a narrative of the findings, we grouped articles into five categories. First, most articles reported on the *correlates of substance use*, representing 22 articles (44 %; Bøhling, 2014, Bøhling, 2015; Carlini et al., 2014; Carlini et al., 2017; Chaney et al., 2019; Erbella et al., 2020; Gilmore et al., 2022; Griffin et al., 2020; Curtis et al., 2017; Droste et al., 2016; Hughes et al., 2019; Hughes et al., 2023; Kaestle et al., 2018; Kelly et al., 2014; Miller, Droste, et al., 2015; Miller et al., 2014; Palamar and Le, 2022; Palamar, Acosta, Sherman, et al., 2016; Santos, Baldin, et al., 2015; Santos, Paes, et al., 2015; Sañudo et al., 2015; Sañudo et al., 2018). Across this diverse set of articles, there were several common outcomes measured, including predrinking, blood alcohol content as measured through biomarkers, risky alcohol use or alcohol dependence, and various illicit substance use. Illicit substance use was most often a general measure, but a few articles examined specific substances. While there was more variability in correlates of substance use examined, they can generally be categorized into a few groups. Demographics were very commonly considered, especially sex, age, and sexual identity. Generally, males had higher rates of substance use in studies. Various aspects of the nightclub environment were also considered, such as the presence of dance floors, crowd composition, promotions (e.g., all you can drink), scene type, and bouncer or police presence. Higher numbers of dance floors and promotions were typically positively associated with substance use outcomes. The time of night was also considered in many studies, finding that perceptions of intoxication vary across the night. While BAC was often an outcome, it is also commonly used as a correlate to examine how it aligns with reported substance or predicts later evening behavior.

Second, 11 articles (20 %; Chan et al., 2016; Jørgenrud et al., 2021; Thurtle et al., 2017; Nordfjærn et al., 2016; Bretteville-Jensen et al., 2019; Palamar, Le, Cleland, et al., 2023; Miller, Curtis, et al., 2015; Palamar and Salomone, 2023; Palamar, Acosta, Calderón, et al., 2017; Waldron et al., 2020; Wieczorek et al., 2021) reported the *prevalence of substance use*. Six of these articles simply reported such prevalence as the main focus of the article, although one of these examined prevalence over time. The remaining five articles examined prevalence by some methodological component in order to examine agreement, such as biomarker and self-report agreement, sampling technique differences, or survey construction choices. We note some of the articles did employ weighting techniques in an attempt to estimate the prevalence of use to the full population of interest.

Third, as an intermediary between correlates and prevalence, we grouped 8 articles (15 %; Cameron et al., 2018; Carvalho et al., 2019; Droste et al., 2018; Hannemann et al., 2017; Mandler, 2016; Palamar, Acosta, and Cleland, 2019; Palamar, Acosta, Ompad, et al., 2017; Palamar, Salomone, et al., 2023) as reporting *patterns of substance use*. Such articles did not formally test correlates, but nonetheless reported group differences in substance use. These differences could be the result of latent class analysis, understanding who under- or overreports substance use relative to biomarkers, or patterns described qualitatively.

Fourth, 8 articles (15 %; Byrnes, Miller, Bourdeau, and Johnson, 2019; Palamar and Le, 2023; Palamar, 2023; Palamar, Acosta, Le, et al., 2019; Palamar, Salomone, et al., 2016; Peacock et al., 2016; Pennay et al., 2017; Sanchez et al., 2015) discussed *harms and safety related to substance use*. Alongside substance use, these articles reported adverse effects, such as accidents, injuries, impaired driving, or unintended substance use. Deterrents, such as group cohesion and prior adverse experiences, were also considered as protective factors.

Fifth, 6 articles (11 %; Baldin et al., 2018; Bourdeau et al., 2015; Byrnes, Miller, Bourdeau, Johnson, et al., 2019; Coomber et al., 2018; Crocamo et al., 2018; Devilly, 2022) described *interventions or legislation related to substance use*. While highly disparate in terms of focus, these articles typically employed a pre/post test or group randomization to test the effect on substance use. Such articles provide experimental or quasi-experimental evidence for enhancing group safety, better transportation choices, nightlife operating hours, and web-based interventions.

## 4 Discussion

This systematic review aimed to explore empirical *in situ* research on substance use in nightlife settings by analyzing 55 *in situ* conducted studies in these environments. By addressing this aim, the review intended to fill a significant gap in the literature and provide insights into substance use in nightlife settings and related harms. To the best of our knowledge, no such review was previously conducted. The results highlight the complexity of substance use patterns, their correlates, associated harms, and intervention efforts within these environments.

As expected, substance use was commonly reported in nightlife settings, with alcohol—including predrinking—being the most frequently used (e.g., Carlini et al., 2014; Sañudo et al., 2018), followed by a wide spectrum of licit and illicit substances, such as nicotine (e.g., Jørgenrud et al., 2021), prescription medications (e.g., Kelly et al., 2014), illicit drugs (e.g., Curtis et al., 2017; Pennay et al., 2017) and NPS (e.g., Wieczorek et al., 2021). Polydrug use emerged as a particularly concerning trend, especially among attendees of EDM events and frequent nightlife attendees (Palamar et al., 2019, Sañudo et al., 2015). Adverse outcomes were especially prevalent among individuals using multiple substances (polydrug use) and those consuming NPS (Palamar, Acosta, Le, et al., 2019). High rates of intoxication-related harms, including driving under the influence, injuries, and unintended substance use, were reported (e.g., Sanchez et al., 2015). Despite the documented harms, only 11 % of studies examined interventions or legislation related to substance use (e.g., Devilly, 2022), and these varied considerably in methodology and outcomes.

The correlates of substance use identified across studies show the following demographic patterns: males, younger individuals, and sexual minorities were more likely to report and test positive for substance use (e.g., Nordfjærn et al., 2016). Studies that focused on LGBTQ+  populations or specific musical subcultures (e.g., EDM or hip-hop venues) revealed distinct patterns of substance use and related harms, often influenced by the cultural context of the venue (e.g., Jørgenrud et al., 2021; Palamar, Acosta, et al., 2016). These populations also reported unique motivations for substance use. Venue-specific characteristics, such as the presence of multiple dance floors, promotional drink offers, and loud music, were also associated with increased substance use (e.g., Carlini et al., 2014, Carlini et al., 2017; Droste et al., 2016). We note inconsistency in reporting of specific sociodemographics as well as the manner in which descriptive statistics are reported (e.g., as categories or central tendencies), hampering cross-study comparison.

As described, however, some studies (20 %) only reported prevalence rates (e.g., Thurtle et al., 2017), without clearly indicating the population represented in their samples. Indeed, we caution against using *in situ* methods strictly for prevalence estimates given that the collection methods make the underlying population hard to identify, although weighting techniques might assuage this concern. On this subject, although there were similarities in study methodologies, they differed substantially in recruitment strategies, ranging from random sampling of venue and/or time (e.g., Santos, Baldin, et al., 2015), and “every X person” technique (e.g., Baldin et al., 2018; Coomber et al., 2018) to no distinctive pattern (e.g. Thurtle et al., 2017; Wieczorek et al., 2021). Thus, there is a considerable mixture of random and non-random methods. While the latter is typical for qualitative studies, we caution against their use in survey research. Studies also differed in reported participation rates, which ranged from 21 % in the study by Carvalho et al. (2019) to 97 % the study by Miller et al. (2014), based on participants approached, not all present at the venue. However, it is important to point out that participation rates were omitted from 21 articles. This variation in recruitment strategies and lack of transparency in participation rates limit reproducibility and the ability to assess sampling biases.

However, a major strength of *in situ* methodologies is the ability to gather real-time data directly from a population that is small subset in general population surveys. This is further advanced if biomarker data are obtained (e.g., breath, oral, hair). Our results show that 56 % of studies collected biomarker data, which provides objective measures of substance use (e.g., Bourdeau et al., 2015; Byrnes et al., 2019). This greatly enhances the validity of self-reported data, which was collected in 93 % of studies (e.g., Palamar, Salomone et al., 2023). The combination of biomarker and survey data offers a more comprehensive and reliable picture of substance use patterns.

The geographic representation of studies was limited, primarily covering the US (e.g., Palamar et al., 2023; Byrnes et al., 2019), Europe (e.g. Bøhling, 2015: Carvalho et al., 2019), Brazil (e.g., Santos, Baldin, et al., 2015) and Oceania (e.g. Droste et al., 2018), and several articles were based on the data from the same studies (e.g. Palamar, Acosta et al., 2016; Palamar, Acosta, Calderón et al., 2017; Palamar, Acosta, Ompad, et al., 2017), which may affect the diversity of findings. The lack of additional countries and world regions may be due to limiting to English language articles or may reflect a lack of *in situ* studies in other locations. We encourage future research examining non-English-language articles as well as studies in more locations, especially where the typical substance used in nightlife may differ.

### 4.1 Limitations and recommendations for future studies

Above, we have noted several limitations to existing studies, many stemming from the considerable variety of research questions examined in nightlife settings and subsequent reporting of methods and results. The variety of research questions among the identified articles means that we did not rate the quality of evidence as some systematic reviews do, as there was no one independent or dependent variable to evaluate and thus making it difficult to compare. Nonetheless, we provided cautions above regarding the use of random methods, reporting of participation rates, and only focusing on prevalence. Future research would benefit from consistency in reporting the recruitment process, participation rates, and compensation for participants to improve transparency and allow comparison. To this end, we recommend reporting guidelines in the form of checklists specific to *in situ* studies, such as STROBE for observational studies (Von Elm et al., 2007) or CONSORT for randomized studies (Hopewell et al., 2025). Having consistent reporting guidelines would allow for stronger evidence, permit reproducibility, and increase transparency. We do not mean to imply that all *in situ* studies should be the same; rather, it would ensure all relevant information is included.

There are several components that emerge from our review. First, authors should report every stage at which selections were made, such as time/venue/district selection, how participants were selected among all participants, how individuals were selected among groups that were selected, how intoxicated persons were identified and removed, who participated among those selected, and how many completed surveys. For any of these stages relevant to a study, a rate should be reported along with any compensation. For the specific case of intoxicated individuals, institutional research review boards will ultimately determine how the research is conducted, but the checklist should include reporting this approved definition. For biomarkers, the checklist should similarly include participation rates, but also reporting the exact method and rates of unusable data. For reporting of descriptive statistics, we recommend the checklist specify exactly how certain data should be reported. As a concrete example, Table 1 shows just how much variability there is in how age is reported, whether as means, standard deviations, ranges, or categories. This list is by no means exhaustive, but rather is illustrative. Our recommendation is for a group of experts to collectively produce these guidelines.

Beyond the recommendation for a checklist, there are other areas for potential future research. For example, we found little use of mixed quantitative and qualitative methods. Further, pairing biomarker data with both quantitative data and qualitative insights could help researchers understand motivations and context of substance use among nightlife attendees. This would allow creation of context specific interventions that would be implemented to support harm reduction for high-risk groups identified in this review, including individuals who use multiple substances (polydrug use) and LGBTQ+  patrons. Furthermore, future research would benefit from exploring longitudinal *in situ* designs, though logistically difficult, to understand causal relationships and long-term effects. By design, we did not include articles that were solely about festivals, as we believe festivals are worthy of their own systematic review given several unique aspects (e.g., all or several day events; availability of drug checking).

Finally, we recognize that this is a very active research area. While our temporal stopping point reflects the work involved in conducting this systematic review, future research should undoubtedly be incorporated as it emerges.

## 5 Conclusion

This systematic review of 55 *in situ* studies underscores both the methodological opportunities and the challenges of conducting *in situ* research in nightlife venues. The value of *in situ* studies is in capturing real-time data on substance use in nightlife settings, including biomarkers, and identifying key demographic, social, and environmental factors associated with elevated risk, thereby providing a basis for harm reduction in these settings. The field would benefit from broader geographical coverage, more intervention studies, and a greater focus on applying findings to practical harm reduction strategies. As nightlife scenes evolve and new substances emerge, continuous and context-sensitive research will remain essential for public health responses. Our findings underscore the importance of culturally sensitive and community-specific approaches in both research and intervention design, that need to combine individual-level strategies with structural interventions at the venue level to effectively address harm reduction.

## CRediT authorship contribution statement

**Mike Vuolo:** Writing – review & editing, Writing – original draft, Visualization, Methodology, Investigation, Formal analysis, Conceptualization. **Renata Glavak-Tkalić:** Writing – review & editing, Writing – original draft, Visualization, Supervision, Project administration, Methodology, Investigation, Funding acquisition, Formal analysis, Conceptualization. **Anja Wertag:** Writing – review & editing, Writing – original draft, Methodology, Investigation, Formal analysis, Conceptualization.

## Funding

This article is prepared as part of the project *Nightlife: A Study in Real and Virtual Context* (REAL NIGHTS), financed by the 10.13039/501100000780European Union – NextGenerationEU (No. 01/08–73/23–2519–13).

## Declaration of Competing Interest

The authors have nothing to declare.
